# Third Dentition

**Published:** 1861-02

**Authors:** 


THIRD DENTITION.—Mr. Carre recently reported, at the Société de Biologie of Paris, the case of a woman, aged eighty-five, in remarkably good health, who after experiencing some pain in the gum had a left upper canine tooth to appear. At intervals of some months, the second incisor on the left side of the upper jaw, and the first bicuspids in the upper and lower jaws, on the right side, appeared.—*Medical and Surgical Reporter.*

